# Confirming the Presence of *Legionella pneumophila* in Your Water System: A Review of Current *Legionella* Testing Methods

**DOI:** 10.1093/jaoacint/qsab003

**Published:** 2021-01-23

**Authors:** James T Walker, Paul J McDermott

**Affiliations:** 1 Walker on Water; 2 PJM-HS Consulting Ltd

## Abstract

Legionnaires’ disease has been recognized since 1976 and *Legionella pneumophila* still accounts for more than 95% of cases. Approaches in countries, including France, suggest that focusing risk reduction specifically on *L. pneumophila* is an effective strategy, as detecting *L. pneumophila* has advantages over targeting multiple species of *Legionella*. In terms of assays, the historically accepted plate culture method takes 10 days for confirmed *Legionella* spp. results, has variabilities which affect trending and comparisons, requires highly trained personnel to identify colonies on a plate in specialist laboratories, and does not recover viable-but-non-culturable bacteria. PCR is sensitive, specific, provides results in less than 24 h, and determines the presence/absence of *Legionella* spp. and/or *L. pneumophila* DNA. Whilst specialist personnel and laboratories are generally required, there are now on-site PCR options, but there is no agreement on comparing genome units to colony forming units and action limits. Immunomagnetic separation assays are culture-independent, detect multiple *Legionella* species, and results are available in 24 h, with automated processing options. Field-use lateral flow devices provide presence/absence determination of *L. pneumophila* serogroup 1 where sufficient cells are present, but testing potable waters is problematic. Liquid culture most probable number (MPN) assays provide confirmed *L. pneumophila* results in 7 days that are equivalent to or exceed plate culture, are robust and reproducible, and can be performed in a variety of laboratory settings. MPN isolates can be obtained for epidemiological investigations. This accessible, non-technical review will be of particular interest to building owners, operators, risk managers, and water safety groups and will enable them to make informed decisions to reduce the risk of *L. pneumophila*.

The recent worldwide COVID-19 pandemic has made many of us reappraise how we manage our water systems and has created a pressing need to address the risk of *Legionella pneumophila* proliferation in unused buildings. As many countries entered lock down, hospitals were busy reorganizing to accommodate the anticipated increase in demand for intensive care units, meaning that some hospital areas were taken out of use temporarily as they were being repurposed. In the UK, exhibition centers were turned into Nightingale Hospitals in a matter of weeks. Buildings in towns and cities were closed as their populations entered lockdown and healthcare disciplines such as dentistry came to a standstill. This meant dramatic changes in the of use of water systems, with the consequent risk of microbial proliferation of waterborne pathogens, in particular *L. pneumophila* (1).

The importance of maintaining management of water systems during the pandemic was highlighted by evidence from China that showed that half of COVID-19 fatalities had experienced a secondary hospital-acquired infection and that 20% of those infected were positive for IgM antibodies for *L. pneumophila* (2, 3). In response to COVID-19, several microbiologists collaborated on European guidance for water safety groups (WSG), responsible persons, and building managers to provide advice to lessen the risk from legionellae^a^ during building closures and their subsequent opening when COVID-19 restrictions were eased. This included guidance for hospitals, other healthcare facilities, and dental surgeries on the precautions that should be applied to minimize risks developing and the tests that should be conducted to provide evidence that water systems are safe to use when buildings are reoccupied (4, 5). This accessible, non-technical review sets out to provide guidance on effective microbiological monitoring for duty holders^b^ and those charged with responsibilities to devise, implement, and manage strategies to control risks from exposure to legionellae arising from water systems. It also aims to provide additional information on effective microbiological monitoring and testing that will enable duty holders, to control risks from exposure of legionellae arising from water systems. In the UK at least, this role is assigned either to multidisciplinary groups, such as WSGs, or an individual, referred to as the “responsible person” (6, 7). The information presented here will inform decisions on testing for *Legionella*, whether applied routinely in the normal course of operations, as part of recommissioning procedures following emergency lockdown of buildings, or in response to other emergency situations, such as a case of or an outbreak of Legionnaires’ disease, where microbiological testing might be required. In this review, the term ‘WSG’ is used throughout and refers equally to situations where oversight of risk management falls to an individual responsible person.

## Legionnaires’ Disease

Legionnaires’ disease is a severe, frequently deadly form of pneumonia that is caused by bacteria belonging to the family Legionellaceae (commonly known as *Legionella*). The family comprises over 60 species and more than 70 serogroups (8), however, the most clinically significant species is *L. pneumophila*. *L. pneumophila* was only identified and named in 1976, but it is now clear that this microorganism was responsible for outbreaks in man-made water systems for a number of decades prior to this (9). In the USA, there has been an almost 900% increase in legionellosis^c^ since 2000 with *Legionella* being the most reported cause of outbreaks of infectious disease linked to drinking water from 2013 to 2014 (10). *Legionella* has also been responsible for the majority of admissions to hospitals (88%) and all deaths associated with outbreaks of waterborne infection associated with drinking water (10). Despite the introduction of legislation and guidance in the intervening years, there has also been an almost year on year increase in Legionnaires’ disease cases in Europe from 2014 to 2018 (11). Over the last five years of data, the notification rates have nearly doubled. Not only were the notification rates for 2018 the highest ever observed for the EU/EEA, but the 2018 data demonstrated that there was a 23% increase in the number of cases compared to 2017 (11).

There are likely to be a number of factors that have contributed to the increased prevalence of Legionnaires' disease, including heightened awareness of the disease leading to increased case reporting, better diagnostic techniques, and also to an ageing population with more people susceptible to infection and with an increasing prevalence of comorbidities (12). It is acknowledged widely that in many countries, the incidences of Legionnaires’ disease cases are underreported e.g., in the USA alone it is considered that only 2.5–4.5% of actual cases are reported to the Centers for Disease Control and Prevention (CDC) (13, 14).

Nineteen of the more than 60 *Legionella* species that have been recorded to have caused as least one infection. However, a single species, *L. pneumophila,* is responsible for 95% or more of Legionnaires’ disease (15–17), of which *L. pneumophila* serogroup 1 is the most virulent. The European Centre for Disease Prevention and Control (ECDC) shows that this has been consistent over time; annual data from patient cultures in 17 countries (*n* = 4719) from 2009 to 2014 identified *L. pneumophila* as the cause of the disease in 97% of cases (18). According to Public Health England(PHE), species other than *L. pneumophila* account for less than 1% of all Legionnaires’ disease cases in England and Wales (19). In Japan the story is similar; 98% of all cases between 2008 and 2016 were caused by *L. pneumophila* (20).

Whilst legionellae are ubiquitous in natural freshwater and anthropogenic water systems, the site source of the infectious bacteria for most sporadic cases, and even some outbreaks, remains unknown (21). However, inhalation of infectious aerosols from contaminated water systems is considered the most common route of exposure and both potable and non-potable systems pose risks. *Legionella* infections have been traced to contaminated water distribution systems (22), showers, faucets, toilets (23, 24), cooling towers (25), and spa pools (26). Evaporative cooling systems, such as cooling towers and related systems, are especially important because of their potential to allow the growth of large numbers of legionellae, if they are not managed effectively, and their potential to transmit infectious aerosols over large distances (27).

## Management and Monitoring of Water Systems

Many people perceive the water supplied by utility companies to be clean and harmless. However, whilst the supplied water is wholesome and safe for drinking, cooking, and bathing, it is not sterile. Proactive water management is needed because even in water systems that are well designed, waterborne pathogens such as *Legionella* can proliferate to an extent that they pose risks to users of the water system, including staff, visitors, and, in particular, certain patients receiving care in a hospital (28, 29). In some water systems, it is inherently difficult to control the growth of legionellae. This is especially true in large buildings with complex water systems, including many hospital buildings, or if water systems are designed poorly, which makes maintaining safe water temperatures problematic. Some buildings have also been modified in ways that exacerbate these problems and some have been constructed using materials that encourage the development of biofilms and provide pockets within the water system where legionellae can thrive.

Because many of the hazards within different buildings and evaporative cooling systems are similar, common principles underpin the various national and international guidelines and standards for water management. These include identification of high-risk areas, maintenance of appropriate temperatures and disinfectant concentrations, system cleanliness, and water turnover. Whatever the specific suite of controls applied to the water system, regular checks need to be made to provide assurances that the controls are effective and risks from exposure to legionellae continue to be managed effectively. As part of the overall management strategy, periodic sampling and testing for the presence of legionellae in water systems can be extremely valuable to determine whether the risk control strategies applied are effective at reducing the microbial risk.

It is important to note that the aim of water management programs is not the complete eradication of all waterborne pathogens. As water entering buildings is not sterile, water management programs are there to identify and prevent/control the *risk* of contracting waterborne diseases (7, 30).

Where people are exposed to environmental pathogens, it could be argued that monitoring of risk reduction strategies should focus on the presence of those specific bacteria that present the highest risk, rather than a wider cross-section of bacteria which may exist within the water system, but present minimal health risk. In the case of *Legionella*, species other than *L. pneumophila*, such as *L. anisa* or *L. bozemani*, may be found present in building water, but figures show that these species represent less than 1% of total Legionnaires’ disease cases (31). Similarly, although a nationwide study of cooling towers in the US documented that 47% of isolates were non-pneumophila *Legionella* species, to date, zero cooling tower outbreaks in the US have been linked to species other than *L. pneumophila* (32). In France, 98% of Legionnaires’ disease cases were attributable to *L. pneumophila* and no other species of *Legionella* have been associated with outbreaks from cooling towers (33, 34). Given the very low prevalence of disease caused by other species, and that less resources should be required to test water solely for *L. pneumophila*, more extensive microbiological testing of water systems could be achieved at the same cost, if a more focused approach to testing is adopted (35).

## *Legionella* Monitoring Policies are Evolving

A number of countries have guidelines or standards to help achieve *Legionella* control. However, the guidance on bacterial monitoring, which most often advocates evaluating the presence and levels of any species of *Legionella* rather than *L. pneumophila*, is based on limited empirical evidence, primarily observations and data gathered during outbreak investigations (36). Not surprisingly, the guidance and regulations vary widely with regard to the location, frequency, and thresholds for action when testing, as well as on the question of what microbiological parameters should be monitored. It should be noted that, while testing requirements vary from country to country, action is always required when *L. pneumophila* is detected at levels above threshold limits (37). Over the last decade, guidelines have continued to evolve, with a shift in parameters from *Legionella* spp. to *L. pneumophila* in France in 2011, and an expanded set of *Legionella* testing method options being accepted by regulatory bodies. The Spanish Standard body recently added PCR to its UNE 100030: 2017 (38). In the UK, the Health and Safety Executive (HSE) recommends that the analysis of water samples for Legionellae should be performed in UKAS-accredited laboratories with the current International Organization for Standardization (ISO) standard methods for the detection and enumeration of legionellae included within the scope of accreditation. The HSE also advises that alternative quantitative testing methods may be used as long as they have been validated using ISO 17994 (39). More recently the UK’s Standing Committee of Analysts added a most probable number (MPN) method to its “Blue Book” for *Legionella* methods (40). The language in the European Drinking Water Directive draft, finalized in 2020 in Brussels, also seems to reflect the changing nature of the field (37). The document directs member countries to implement a risk-based approach and cites the ISO 11731:2017 method for minimum testing requirements. It also states that for risk-based verification monitoring, and to complement traditional culture, other methods, such as ISO/TS 12869, rapid culture methods, non-culture-based methods, and molecular-based methods, in particular qPCR, can be used (37).

Evidence following the change in the French national policy suggests that implementing control measures both for cooling towers and hospital water networks, and focusing specifically on *L. pneumophila,* can be an effective and cost-effective strategy to control Legionnaires’ disease (41). Reports have indicated that nosocomial cases have decreased from 20 to 5–6% and that the percentage of these cases in comparison to the total number of explained cases has decreased from 33% to around 20% (1998 to 2018) with a lack of outbreaks in the last decade (42, 43).

Risk reduction strategies that focus on the presence of specific species of bacteria that present the greatest risk is not unprecedented. For example, *Pseudomonas aeruginosa* is routinely tested for in hospital augmented care areas to assess the efficacy of control measures and risk levels, rather than monitoring all species of *Pseudomonas* (35), despite the fact that other species of *Pseudomonas* cause infections, albeit rarely (44). Likewise, testing for *L. pneumophila* rather than other species within the genus identifies the presence of the most significant pathogen whilst also providing an indication that conditions exist within the water system being monitored that could allow other pathogenic strains to grow. As is the case when testing for *P. aeruginosa*, detecting *L. pneumophila* should lead to prompt actions be taken to reduce the risks of exposure to legionellae.

## Historical Perspective of Microbiological Investigations

One of main challenges of environmental testing to assess risks from Legionnaires’ disease has been the ability to detect *L. pneumophila* consistently, given the limitations of traditional methods. In 1976, over 5000 microscopy tests and 14 different types of bacterial culture media were developed in attempts to grow the organism that had caused the outbreak of pneumonic disease during the annual convention of the American Legion at the Bellevue Hotel in Philadelphia (9). However, isolation of the microorganism was no easy task and eluded CDC scientists until early 1977 when the organism was finally isolated and named *Legionella pneumophila*. “*Legionella*” in honor of the American Legion victims and “pneumophila” after the acute pneumonia caused by the bacterial infection. The plate culture media that were developed then were specifically designed to grow *L. pneumophila*. Those media have been refined in the intervening years, but are still primarily selective for *L. pneumophila*, though other species can be isolated if sufficient numbers of colonies are selected for identification. A particular issue with these selective media is that they are not absolutely selective, such that agar plates can be overgrown with other, faster growing microorganisms which can obscure colonies of *L. pneumophila* if they are also present in the water sample that is being tested. This can be a particular problem when legionellae arepresent in water samples at low levels. The identification of single colonies may be difficult without a considerable amount of prior experience by the laboratory analyst (45). This creates the potential for false negative results and therefore a false sense of confidence for the WSG charged with managing the risks in a facility.

Due to the inherent problems in isolation media for clinical diagnosis of Legionnaires’ disease, guinea pigs were used until 1983 for the isolation of legionellae from autopsy and other clinical specimens. This, as much as anything, drove investigators to develop more sensitive and selective nutrient media (46, 47). The development of glycine, vancomycin, polymyxin, and cycloheximide (GVPC) media was designed to suppress the growth of accompanying microorganisms (48–50).

The isolation of legionellae from environmental samples was particularly challenging because the bacteria may be present at low levels, requiring water samples to be filtered (or centrifuged) in order to concentrate the number of bacteria from the sample and improve recovery.

In attempts to address the problem of plate overgrowth by faster-growing commensal bacteria, researchers exploited the enhanced survival rates of legionellae in acidic conditions (pH 2.0) and also their relative heat tolerance (able to withstand temperatures of >50˚C) (51–54). These acid-tolerant and heat-tolerant traits were used in the development of pre-treatment steps in the laboratory procedures for the isolation of *L. pneumophila* on buffered charcoal yeast extract (BCYE) and GVPC agar plates. However, not all species of legionellae are recovered equally using these pre-treatments and Dennis et al. (54) noted that whilst *L. pneumophila* had a decimal reduction time of 111 min in water at 50˚C, its related species, *L. micdadei,* had a decimal reduction time of between 2.4–7.5 min, meaning that the latter would be less likely to be recovered using this methodology. The original 1970s methods and these pre-treatment steps that were developed in the 1980s, are still included in the internationally recognized standard for the enumeration of legionellae (ISO 11731: 2017). Consequently, particular acid- and heat-tolerant species of legionellae, such as *L. pneumophila*, are positively selected and other, less tolerant species are likely to be inhibited during the pre-treatment stages and will not be detected on agar plates.

Limitations of ISO 11731:2017 are acknowledged in the scope of the standard, which clarifies that the methods described in this document do not recover all species of *Legionella*, and that plate methods may recover various species but cannot be counted on to reliably detect all species of the bacteria. Whilst *L. pneumophila* (and some other species) is relatively tolerant to pretreatments, there will still be some loss of viability, with fewer colonies detected on agar plates than if the pretreatment steps were omitted. It should also be noted that some loss of recovery of legionellae, including *L. pneumophila*, can be expected during the filtration (or centrifugation) steps that are applied.

The deficiencies of the standard method have been acknowledged previously and researchers from the New York City Department of Health made reference to the risk of not detecting *L. pneumophila* when using the standard plate method only. This resulted in an amendment for cooling tower outbreak investigation protocols to require the use of both the traditional plate and the MPN culture methods to “maximize the likelihood that Lp1 (*L. pneumophila* serogroup 1) could be isolated from a given water sample” (55).

Despite these and other drawbacks, the traditional plate culture method is often referred to as the “gold standard,” but it could be argued that it is far from that. Among the criticisms of the method is the lengthy incubation period of 10 days, which introduces a significant delay in obtaining results. This delay can be of great importance in outbreak investigations when information on the safety of potential sources of infection is needed urgently. In addition to outbreak investigations, shorter reporting timeframes are also valuable for routine monitoring activities to provide assurances that any remedial actions taken have been effective.

The shortcomings of plate culture have led to several innovative approaches to detecting and, in some cases, attempting to enumerate legionellae in water samples. All methods have a role to play in *Legionella* risk management strategies. Which one is used in preference to another will depend on the purpose of the testing and the timeframes in which the results must be reported.

## Microbiological Methods

Microbiological testing is an important element of the overall monitoring program and there are a number of recognized methods available for detecting legionellae in water systems compared to a decade or so ago. Each has relative merits and potential drawbacks, so the question is; how does a WSG decide on the most effective testing method? In this paper, we review a number of microbiological methods that are currently available and discuss their relative advantages and disadvantages.

## Plate Culture

There are a number of advantages in using plate culture including, (i) the ability to compare results with those from historical samples analyzed using the same method, (ii) growth of viable cells provides a means of quantifying the numbers of legionellae present, and (iii) it can be used to recover clinical and environmental isolates that can then be used to determine whether there is a link between a water system and a case of infection. These are valuable traits, but disadvantages also exist for the standard method.

Plate culture methods, such as those published in ISO 11731; 2017 are still the most commonly used for environmental surveillance of *Legionella*. This method estimates the number of legionellae in water samples, presented as colony-forming units (CFU) per unit volume of water sampled, and is usually expressed as CFU/L. The method includes potable, industrial, waste, and natural water samples and involves the collection and transportation of (usually) 1 L aliquots of water (56–58). Depending on the water matrix to be processed, laboratory personnel must choose from four procedures, four treatments, and four selective culture media. This means there are 14 possible procedural scenarios for each sample if the ISO 11731 (2017) procedure is used (57).

It is worth noting that, in the national foreword to the UK’s publication of the ISO standard (BS EN ISO 11731: 2017 (106), the British Standards Institution (BSI) commented that it had voted against the approval of ISO 11731 (2017) as a European Standard. Although BSI was obliged to publish the standard, it cited, amongst other things, potential variations and interpretations, not only between the method options, but also within each method, that would lead to different approaches being taken by different laboratories and that this could yield different results.

Uncertainty in inter-laboratory precision and accuracy of plate methods for *Legionella* detection is well recognized (59). In the UK, PHE administer an external quality assessment *Legionella* isolation scheme to provide checks on the consistency of results produced by different laboratories that examine water samples for the presence of legionellae (60). In the USA, to ensure laboratory capacity for outbreak investigations, the CDC felt the need to establish the Environmental *Legionella* Isolation Techniques Evaluation program that enables laboratories to evaluate their *Legionella* isolation techniques by using standardized, blind samples and is based on semi-annual proficiency testing (45).

Whilst the culture plate method has been refined since the 1970s, and more options provided, the basic process remains remarkably unchanged. The complexity and multiple numbers of plates that are built into the procedure are required because the approximate number of legionellae in any given water sample is usually unknown. Given this, the analysts undertaking these tests must use any additional information that accompanies the water sample, combined with their own knowledge and experience of water systems, to select and run the procedure which will give the best chance of growing countable numbers of colonies on the culture medium without interference from other commensal microorganisms.

In addition to the problems that fast-growing commensal microorganisms present in obscuring colonies of legionellae on agar plates, there are issues regarding the number of colonies required on each plate to provide statistical validity to the enumeration process. According to BS EN ISO 11731 (2017), the range varies according to whether one is counting a single strain of *Legionella* (10–300 CFU/plate), or whether there are interfering microorganisms present (10–150 CFU/plate) and where the membrane filter technique is used (10–80 CFU/filter). So, the many different ranges to count has the potential to introduce further variation in the interpretation of results.

Filters can present further problems, especially when filters are placed on the agar surface. This is because colony sizes can vary and the much-reduced surface area of the membranes compared to that of a standard plate can make counting colonies that develop and overlap problematic (61, 62).

Laboratory analysts must be skilled and experienced in identifying colonies of legionellae and must use their discretion when examining a large number of plates, some of which may be overgrown. Such individual interpretation of counts, as well as the identification of isolated colonies, introduces elements of subjectivity to the process (61, 63, 64).

As has been said, legionellae grow very slowly on solid growth media, and whilst the ISO standard does not specify a definitive laboratory incubation time for plates, a range of between 7 and 10 days is given. In practice, most laboratories incubate plates for at least 10 days. For the CDC and Standard Methods, a minimum of 9 days is required for presumptive^d^ colonies to be confirmed with BCYE or blood agar plates.

Further delays in receiving confirmed results from sampling are introduced by transportation, accession of samples, reading plates, and then reporting the results, depending on the laboratory performing the analysis. This means that results from testing may not be available for 2 weeks and the WSG may have to delay important decisions, such as whether it is necessary to implement decontamination strategies for water systems or parts of them.

Other limitations of the plate culture method include its inability to recover legionellae in the viable but non-culturable (VBNC) state, the significance of which, in relation to the likelihood of VBNC cells to cause infection, remains uncertain (65). As previously suggested, plate culture agars have been optimized to select for the growth of *L. pneumophila*, and as such other species which only rarely cause disease may not be recovered (66). There are also costs associated with collecting (often 1 L) and transporting large numbers of samples to laboratories that may be significant distances away (67). Studies suggest that sending samples to external laboratories can introduce further inaccuracies and influence test results due to transportation times and conditions (68). Although other studies have not found a statistically significant difference with holding times of up to 72 hours, processing samples as soon as possible is good practice (67).

## Most Probable Number Methods

MPN quantification is a liquid culture method for the selection, identification, and quantification of bacteria and one system has been developed for the detection of *L. pneumophila* in water samples. Sample preparation for potable waters is simple and straightforward and does not require high temperature pre-treatments, or concentration steps, such as centrifugation or filtering. When processing non-potable waters, a brief pre-treatment step is required. The method uses a powdered growth medium, to which a measured aliquot of the sample is added. The reconstituted medium contains a defined growth substrate that selects positively for the growth of *L. pneumophila* only and suppresses the growth of other commensal microorganisms that might be present in the water sample. Enumeration is achieved by placing 100 mL of the prepared sample/medium solution into a sealable quantification device which is then incubated at 39°C ± 0.5°C or 37°C ± 0.5°C for potable and non-potable samples, respectively. Once the quantification device is sealed and after a 7-day incubation, *L. pneumophila* can be enumerated by counting those cells demonstrating growth (indicated by a change in color and/or turbidity of the growth medium) and determining the number present by consulting an MPN lookup table.

The advantages of the MPN method include the ease with which the tests can be performed and the unambiguous results that are obtained (69, 70). The single protocol per matrix [rather than the multiple potential routines offered by ISO 11731 (2017)] for potable water alone and the reliance on a simple determination of a binary color/no-color result, rather than subjective identification of colonies by different analysts, reduces the measurement uncertainty obtained from MPN results. This consistency is likely to provide advantages for WSGs or others who have responsibilities for water systems that use *Legionella* trending data to make water safety decisions. In addition, MPN testing provides confirmed numbers of *L. pneumophila* in the sample at 7 days, which can expedite actions that might need to be taken based on test results. Because MPN is a culture-based method, viable bacteria can be recovered from positive wells in the blister pack and cultured further if required for investigational purposes during single case or outbreak investigations. However, this could be considered a limitation as it does require an additional culture step.

Laboratory studies have shown that the MPN method generates higher counts, on average and has a higher specificity for *L. pneumophila* (97.9%) in comparison to the extant ISO 11731 standard method (95.3%) (69, 71). The higher counts may be due to improved recovery rates of *L. pneumophila* in a liquid growth medium compared to an agar medium, as has been demonstrated for other bacteria (72) and/or to the fact that bacteria are not damaged by filtration or centrifugation steps. Importantly, from an infection risk control perspective, studies have demonstrated equivalence in results from the MPN assay and the ISO standard in terms of CFU/L. This means that MPN results can be applied directly to national action levels, allowing the implementation of appropriate remediation measures to ensure the ongoing safety of water systems (39, 71, 73).

In some applications, e.g., for water samples that have high numbers of non-*Legionella* organisms, such as those from cooling towers, MPN has been shown to have several advantages over the plate method, with a significant increase in sensitivity for *L. pneumophila* (74).

Another potential attraction of the MPN method is that the test is simple to perform and requires little in the way of specialist laboratory equipment. This introduces the possibility of on-site sampling and testing by suitably trained and competent operatives, for example in a hospital setting. This type of arrangement would reduce further the overall reporting time, as it eliminates time taken for transportation of samples to, and to receive results from, a third party testing laboratory. As with any analytical testing, whether on site or off site, some form of ongoing quality assurance scheme is advantageous, as is appropriate accreditation of the method in any third party laboratory performing the testing.

Perhaps of less significance, but worthy of mention, is that the available MPN method processes water samples of 100 mL (compared to 0.5 to 1 L samples required for solid medium tests), so sample transport costs and the environmental impact of transportation are likely to be lowered.

The MPN method only detects *L. pneumophila* and other legionellae will not grow in the liquid medium. This might be perceived as a disadvantage of the method and it would be if a particular sampling strategy sought to detect and count only non-pneumophila species. However, given the clinical significance of *L. pneumophila*, discussed earlier in this paper, such a strategy for routine monitoring of control measures seems unlikely. Indeed, it could be argued that detecting *L. pneumophila* in a water sample could be taken as an indication that conditions exist within the water system, from which the sample was derived, that are conducive to the growth of other species of legionellae, as well as *L. pneumophila*. Given the improved results compared to those of standard plate culture, MPN could be a useful tool in determining whether such conditions existed.

Assurance that the MPN method is at least as effective in detecting and enumerating *L. pneumophila* in water samples as the standard plate count methods can be gained from the UK’s Standing Committee of Analysts’ recent update on determining the presence of *Legionella* bacteria using MPN alongside the plate count method (40). Inclusion in the “Blue Book” indicates that the MPN method has been fully validated for the detection of *L. pneumophila* from potable and related water systems (40).

## Polymerase Chain Reaction

PCR was first developed in the 1980s and since then the technique has been developed and applied in a wide range of applications. More recently, it has been used for the detection of legionellae in water systems and in clinical specimens taken from patients suffering from Legionnaires’ disease (75–77).

To begin the analysis, environmental water samples are first collected and filtered. The bacteria are eluted from the filter and lysed in an appropriate buffer solution, and the DNA is extracted and used for amplification and quantification. PCR works by cycling between high and low temperatures to separate (denature) DNA in the sample and then attach (anneal) DNA molecules called “primers” that are specific for the target DNA and which direct an enzyme called DNA polymerase to copy the target gene sequence. At each round of heating and cooling (a thermal cycle) the amount of target DNA in the sample is amplified exponentially until it reaches a point at which it is measurable using fluorescent dyes. With quantitative PCR (qPCR), the amount of target DNA produced following a given number of thermal cycles can be used to determine how many copies of the DNA (and hence an indication of the number of bacteria) were present in the original water sample and provide a result expressed in genome units (GU).

A number of publications have described the use of qPCR methods for the detection of legionellae in water samples and a number of commercial *Legionella* PCR systems are available including an on-site qPCR detection system which the manufacturer claims is easy to carry out and provides results in less than one hour (75, 77–81).

The high reproducibility and quantitative aspects of qPCR has seen it accepted by the Association Française de Normalisation (AFNOR) and ISO as a standard method for the detection and quantification of *Legionella* spp. and *L. pneumophila* (standards NF T90-471 and ISO/TS 12869:2012, respectively) (73, 107). The PCR method described in ISO 12869:2012 is also referenced as an alternative method in the 2020 European Drinking Water Directive text. However, national and international guidance cites action limits for the control of *Legionella* in CFU and there is currently lack of agreement in the scientific community on the interpretation of genome units in comparison to CFU.

The main advantages to qPCR, over plate culture and MPN, are rapid turnaround times and improved sensitivity/specificity. In particular, samples can be processed and results reported within 24 h. In comparison to plate culture and qPCR, Collins *et al*. demonstrated that PCR had a 100% negative predictive value (NVP), i.e., a sample in which legionellae is absent will always test negative by qPCR compared to culture (82). In addition, qPCR was the only method able to detect *Legionella* in a fatal case of Legionnaires’ disease associated with a hired birthing pool. Direct sequence-based typing on the qPCR positive samples provided epidemiological typing data to assist investigations (83).

One of the anomalies of PCR is the increased number of positive samples in comparison to culture (84). Whiley et al. aggregated the results of 28 studies and reported that 72% (2856/3967) tested positive for the presence of *Legionella* spp. using qPCR and 34% (1331/3967) using culture (84). Such anomalies may be accounted for by the presence of DNA from damaged, stressed, or VBNC cells present in a sample, particularly in chemically treated water, which can make assessments of the effectiveness of control or remediation efforts to reduce bacterial numbers problematic (85, 86). To address the difficulties caused by damaged cell and/or VBNC legionellae, qPCR methods have been developed that incorporate the use of ethidium or propidium monoazide (87–89), which render cell-free DNA unavailable to detection by the PCR reaction chemistry. However, whilst methods to assess viability are now available, they currently lack standardization. This means that, currently, a degree of uncertainty remains in the results of analyses.

PCR has now been around for several decades and has been proposed as the definitive assay that will provide answers to the key questions about the presence of *Legionella* in water samples and was projected to become the dominant test. To date this has not happened, and the plate culture assay is still the historically accepted method of choice. In the UK, plate culture was used during the investigation of the “Heads of the Valleys” outbreak in Wales in 2010 and PCR was used to complement the results from plate culture. However, there was a lack of interpretation and understanding between genome units and CFU which caused confusion and concern, as the PCR results indicated a number of positive samples that subsequently tested negative by culture (90). As a result, there was a lack of confidence in PCR even as an investigative tool and, consequently, many water system managers continue to use the old historically accepted plate culture processes. And of course, not all water laboratories have the molecular microbiology testing facilities, equipment, financial resources, or trained staff for routine molecular analyses (91).

## Immunomagnetic Separation (IMS)

Immunomagnetic separation (IMS), a technique that uses small super-magnetic particles or beads coated with antibodies against surface antigens of bacterial cells, has been available since the early 1990s (92, 93). It provides a simple but powerful method for specific capture, recovery, and concentration of the desired microorganism.

The *Legionella* detection system is based on IMS that uses anti-*Legionella* species immune-modified magnetic beads coupled to enzyme-linked colorimetric detection (64). In principle, the original water sample is concentrated by filtration according to ISO 11731 (2017) and the cells eluted and analyzed. A suspension of anti-*Legionella* species immunomodified magnetic beads is added. Where cells of legionellae are present in the prepared sample, they bind to the antibodies immobilized onto the surface of the magnetic beads to form bacteria/bead complexes. The complexes are then separated by a magnet field, washed, resuspended, and then incubated with a horseradish peroxidase (HRP)-conjugated anti-*Legionella* antibody to form labeled complexes which are visualized by the colorimetric reaction developed when HRP substrates are added.

There are several advantages of the IMS technology including high sensitivity and specificity (96.6 and 100%, respectively) with a reported efficiency of 97.8% when compared against culture (ISO 11731: 2017) (57). The false positivity has been reported to be 0% and the false negative value reported as 3.4% (94). In a comparative trial of culture, IMS, and PCR, Diaz-Flores et al. reported that *Legionella* spp. were detected by culture in seven (25.9%) of 27 samples and 18 (66.7%) of the 27 samples were positive by the IMS method (64). Other advantages to the IMS methods include rapid analysis which, because the method does not rely on the growth of legionellae, can be available in a few hours even in heavily contaminated waters and in the presence of growth inhibitors (64). Hence, IMS appears to be particularly useful in the early identification of potential risk sources in a Legionnaires’ disease outbreak, for rapid implementation of interventions (95). Manufactures also claim efficient detection of multiple species of legionellae in water samples, although it is not clear precisely which species are detected and which are not.

Despite the perceived benefits of IMS assays, they have not yet been adopted widely by the scientific community or testing laboratories. Whilst the manufacturers claim that this method is simple and easy to perform, their instructions include many individual steps. However, they also claim that experienced laboratory technicians can undertake the test in one hour with batches of tests being run simultaneously.

The manufacturer has announced recently that automated methods are now available and a fully automated proces is now claimed (96).

Whilst the anti-bodies bind to antigens in the cell wall it is feasible that the magnetic beads may attach to and detect VBNC and damaged cells or fragments of cell walls, and as such may overestimate detection rates in comparison to culture-based methods. The manufacturer provides a conversion formula so that the colorimetric signals produced by IMS methods, combined with photometer readouts, can be described in “equivalent colony forming units” (CFUeq) enabling some reference to national action levels (95, 96).

## Lateral Flow Technologies

Devices based on lateral flow (LF) technologies were developed initially for clinical diagnostic purposes to detect *L. pneumophila* antigen, which is excreted in the urine of patients suffering with Legionnaires’ disease; the urinary antigen test. A limitation of most commercially available clinical diagnostic tests is that they can only detect *L. pneumophila* serogroup 1 antigen, meaning that other serogroups of *L. pneumophila* (and other species within the genus) that could be the cause of disease, would be missed. More recently, LF has been adapted for testing environmental water samples, but as with the clinical tests, only *L. pneumophila* serogroup 1 is detected.

The LF detection assay uses a plastic paddle in which capillary flow technology binds a colored antibody to any *L. pneumophila* serogroup 1 antigen that is present in the sample. When the sample is positive, this is indicated by two red lines; one line to show that the test has been completed successfully and the other to indicate the presence of antigen. In a number of assays, the sensitivity has been shown to be too low for the determination of *L. pneumophila* serogroup 1 in some environmental water samples (97, 98). However, the LOD can be improved and is reported to be approximately 100 CFU/L when an additional filtration step is implemented (99).

Nonetheless, there are several advantages to LF devices, including the reduced time taken (under an hour) for a positive result, that the test kits are small, lightweight, portable, easy to use on site for all types of water, and that interpretation of the result is visual such that specialist personnel and laboratory infrastructure is not required.

As with any test, LF has a number of disadvantages; the test in not quantitative and so only provides an indication that *L. pneumophila* serogroup 1 is either present or absent in the water sample tested (99). Where a quantitative result is required or where there is a need to detect other species of *Legionella*, or serogroups of *L. pneumophila*, then other tests would need to be performed.

## Discussion

The World Health Organization, ECDC, and CDC have clearly identified *Legionella* as the number one microbial pathogen in water systems for more than a decade (15, 17, 100). Many countries have implemented regulations, guidance, and standards to assist in the control of waterborne pathogens and in particular *Legionella* spp. Yet outbreaks continue to occur (101–104). While researchers have identified more than 62 species of *Legionella*, *L. pneumophila* is responsible for >94% of the culture-confirmed Legionnaires’ disease cases notified in 2018 in the EU/EEA (European Legionnaires’ disease Surveillance Network annual meeting 2019, unpublished data) and non-pneumophila strains only account for <1% of clinical cases in England and Wales (19).To reduce the risk of Legionnaires’ disease, WSGs and responsible persons need to ensure that water management strategies are effective in controlling the risks that cause Legionnaires’ disease, namely the presence of *Legionella*. Hence, should routine inspection and microbiological monitoring be implemented to assess the presence of the most pathogenic species, namely *L. pneumophila*, instead of identifying a range of *Legionella* species, almost all of which represent a much lower health risk than *L. pneumophila*?

There is now a range of commercially available tests that WSGs can select to undertake microbiological testing of their water system to assess risk control strategies for legionellae, including the historically accepted plate culture, the MPN assay, PCR, IMS assay, and LF devices. The appropriate choice will depend on the WSG’s priorities and the purpose of the testing that is undertaken. WSGs will need to consider whether:

- Testing is required purely for routine monitoring of controls to proactively ensure risk is managed.
- Parts of the water system harbor *L. pneumophila* at undetectable levels.
- Longitudinal and consistent data are required for trending purposes.
- More sampling is required e.g. more frequently or at more sites.
- There are concerns about resources or sample collection and transport logistics.
- There are benefits from having testing done on site.
- There is a benefit from quicker return times for test results.

Some considerations are captured in Table 2, including rapidity of testing, sensitivity, specificity, quantification, quality assurance, and validation of the assay. Different approaches can be taken depending on the WSG’s priorities.

**Table 1.** Methods used for the detection of *Legionella* from water samples

Test method	Plate culture	MPN	qPCR	IMS	LF
Time to results	7–14 days	7 days	Same day	Same day	Same day
Presence/absence	Yes	Yes	Yes	Yes	Yes
Quantification	Yes (CFU/volume)	Yes (MPN/volume)	Yes (GU/volume)	(CFUeq/volume)	No
Live or dead cells	Live	Live	Detects DNA from all cells	Live and potentially dead/damaged cells	Live and potentially dead/damaged cells
Detect VBNC	No	No	Yes	Yes	Yes
*Legionella* spp.	Yes	No	Yes	Yes	—
*L. pneumophila*	Yes	Yes	Yes	Yes^a^	Yes
Isolate available	Yes	Yes	No	No	No
Sensitivity	Low (105)	98%	Sensitivity is better than culture methods	95.3%	100 CFU/L
Specificity	95.3%	>97.9%	100%	88.4%	Unknown
LOD	1 CFU/100 ml (76)	≥1 organisms/100 mL	480 Genome Units (GU) per litre (L)	Equivalent to culture	Unknown
False positive	83% (68)	<4%	Yes—but lower if free DNA removal solutionused	11.6%	Unknown
False negative	74% (68)	4.2%	No—high negative predictive value	4.7%	Unknown
Validation	Comparison against other techniques (57, 69)	Comparison according to ISO 17994 against ISO 11731:2017 (39, 57) Equivalence according to (73) and approved and accepted in the SCA “Blue Book” (39) AFNOR validation (73)	NF validation; NF T90-471; ISO/TS 12869 (73, 107)	Certified by the AOAC Research Institute Meets the standard ISO-17381 Meets the requirements in Spain’s UNE100030 guidelines (38, 108)	Unknown
Sample preparation	Yes	Only for non-potable samples	Yes	Yes	Yes
On-site test	No	Yes^b^	Yes^c^	Yes^b^	Yes
Laboratory test	Yes	Yes	Yes	Yes	No
Routine monitoring	Routine	Routine	Routine	Routine	Routine
Specialist expertise required	Yes	No	Yes	Yes, training provided	No
Advantages	Compares with historical samples Accepted measure of viability and quantification by regulators and standard bodies Recovery of isolates for epidemiologic investigations	Rapid sample preparation and processing Requires ≤100 mL No pretreatment step for potable samples No specialist laboratory required High sensitivity and specificity for *L. pneumophila* Isolates can be recovered	High specificity, sensitivity Rapid assay Low detection limits and the possibility to quantify the concentration of the microorganisms in the samples using real-time PCR	Same day sample processing to result	Small, rapid, portable, no training required, easy interpretation
Disadvantages	Length of time for results (7–14 days) Low sensitivity Loss of viability of bacteria after collection, difficulty in isolating *Legionella* in samples contaminated with other microbial (92) Presence of interfering microbiota and the inability to detect VBNC state (65) Both accompanying organisms and inhibitors may cause a rate of inconclusive results (64) https://www.ncbi.nlm.nih.gov/pmc/articles/PMC4436101/ Designed for *L. pneumophila* so does not recover all species of *Legionella* Interpretation of isolates on agar can be problematic (57) Inter‐laboratory variations have been reported Collection of 1 L samples is cumbersome and transportation costly Pretreatment steps required Carried out by specialist laboratory	Length of time for results 7 days Inability to detect VNBC Detects only *L. pneumophila*	Requires sophisticated and expensive equipment, appropriate specialist installations, and trained personnel PCR inhibiting compounds present in environmental samples may cause false negatives False positives can be caused by the inability of PCR to differentiate between cells and free DNA	Manual method requires trained staff	Need high presence of *L. pne* *umophila* Presence/absence only
Key publications	(57)	(34, 61, 69, 71, 74)	(75, 76, 78–82, 89)	(64, 92–94)	(97, 98)

a Does not distinguish between *L. pneumophila* and *Legionella* spp.

b On site in this context refers to in-house testing without the need for a standard microbiology laboratory.

c At the time of writing this applies to one method.

**Table 2.** Considerations for method choice

Test method	Plate culture	MPN	qPCR	IMS	LF
Presence/absence	Yes	Yes	Yes	Yes	Yes
Quantification	Yes (CFU/volume)	Yes (MPN/volume)	Yes (GU/volume)	Yes (CFUeq/volume)	No
*Legionella* spp.	Yes	No	Yes	Yes	No
*L. pneumophila*	Yes	Yes	Yes	Yes	Yes
On site	No	Yes^a^	Yes^b^	Yes^a^	Yes
Laboratory	Yes	Yes	Yes	Yes	No
Same day	No	No	Yes	Yes	Yes
7 days	>7 days	=7 days	<7 days	<7 days	<7 days
8–14 days	Yes	<8–14 days	<8–14 days	<8–14 days	<8–14 days

a On site in this context refers to in-house testing without the need for a standard microbiology laboratory.

b At the time of writing this applies to one method.

### Is the result required that day?

Where results are required the same day, the currently available choices appear to be either LF, IMS, or PCR. LF is quick and easy to undertake on site by non-specialists and gives a qualitative (i.e., non-quantitative) presence/absence determination with results dependent on the number of legionellae cells present, with positive results more likely to be obtained from a water system where contamination would be at higher concentrations, such as cooling towers, rather than from a potable water network. For semi-quantitative analyses, PCR could be selected with samples typically forwarded to a specialist laboratory and results reported within 24 h.

### Can the sample be processed on site?

All WSGs are likely to be evaluating the best use of their finite resources, whether it be to optimize the number of samples analyzed or to simplify logistics. WSGs may value an internal chain of custody, in which case, on-site testing and the ability to use in-house staff, allowing greater flexibility for sampling and timing of analyses, could be considered advantageous. For those that wish to carry out analyses on site, then the LF, some PCR methods, and MPN methods could be undertaken by non-specialist, but suitably trained, personnel.

### Is a quantitative result required?

National regulatory and guidance standards set action levels to provide recommended actions to be taken depending on numbers of legionellae that are detected in a water system (39). Where a direct (i.e. culture-based) quantitative test is required, then either plate culture or MPN can used. PCR or IMS can provide indirect (i.e., non-culture based) quantitative results. It should be remembered that action limits are stated in units derived from culture-based methods. Therefore, CFU per volume or MPN per volume may be required, although CFUeq might be considered acceptable. Reliable quantitative results are also needed for WSGs looking for unexpected or unacceptable increases in *Legionella* numbers. WSGs need consistency in test results so that they can be confident that changes in the number of legionellae detected correspond to actual changes in their water system rather than to the inherent variability in the detection method used. In this case, among these options, a simple quantitative test with high repeatability, such as MPN, may be desirable. MPN will provide a quantitative result for *L. pneumophila*, has a higher sensitivity for potable water, and is equivalent in terms of CFU/L when compared to plate culture (35, 71, 73, 105).

### Is there a need to respond to an identified problem from legionellae other than L. pneumophila?

If the WSG is undertaking routine monitoring for a facility where non-pneumophila *Legionella* spp. have previously caused infections or could pose a risk to, for example, high-risk patients in hospitals, either plate culture or PCR can be used to identify these less common *Legionella* species. They would also be detected by IMS, although this would not provide speciation. Neither LF nor MPN would detect non-pneumophila legionellae. The WSG should of course use caution when assessing the results for non-pneumophila *Legionella* spp. This is because the presence or growth of non-pneumophila species in a water system could indicate that suitable growth conditions for *L. pneumophila* have at some point been achieved. As such, these organisms should be seen as indicators of system colonization and appropriate action taken on their detection.

#### Summary

By considering carefully their needs and priorities, WSGs can make informed decisions about testing approaches and methods which best help them achieve their goal of reducing Legionnaires’ disease risk. The plate culture method is a historically accepted technique but has issues of recovery due to overgrowth and requires highly specialist facilities and equipment and highly trained personnel. Interpretations of plate counts can be subjective and introduce test result variability that is unrelated to the facility water quality. PCR is sensitive, specific and, like the plate count method, has been accepted by international standardization bodies. It is rapid, providing results in less than 24 h, but concerns remain over interpretation and reporting numerical results for use with published action levels. IMS technologies are able to provide results within 24 h and whilst the manual method requires a number of different steps there is now an automated process. LF devices provide rapid presence/absence results but only where sufficient legionellae are present in samples. In comparison to the other methods, MPN provides a simple, robust, and reproducible process that is validated and recognized by standardization bodies and can be used on site as well as in the laboratory setting where accreditation and external quality assessments can be undertaken. MPN detects only *L. pneumophila*, but with greater sensitivity than the plate count methods and so has the potential for use as a powerful monitoring tool that can be used in conjunction with action levels in approximately half the time required for plate culture.

## Endnotes

a Legionellae is the plural trivial name used to describe all bacteria belonging to the *Legionella* family.

b A person or organization that is in control of premises in connection with a trade or business.

c Legionellosis is the collective term used to describe illness due to infections caused by *Legionella* and include Legionnaires’ disease and the less severe, non-pneumonic Pontiac fever and Lochgoilhead fever.

d Morphologically distinctive bacterial colonies can usually be detected within 3 to 5 days and identified presumptively as *Legionella* species prior to identification being confirmed by specific immunologic typing of the isolated bacteria or by molecular analysis.
